# Atypical presentation of varicella-zoster virus reactivation in a lung transplant patient: a case report

**DOI:** 10.1099/acmi.0.000763.v3

**Published:** 2024-07-11

**Authors:** Ingrid Hoff, Eivind Rath, Slobodanka Pena-Karan, Elisabeth Sivy Nginamau, Are Martin Holm, Turid Thune, Tehmina Mustafa

**Affiliations:** 1 Department of Microbiology, Haukeland University Hospital, Bergen, Norway; 2 Department of Medicine, Telemark Hospital Trust, Porsgrunn, Norway; 3 Department of Medicine section of Infectious Diseases, Haukeland University Hospital, Bergen, Norway; 4 Department of Emergency Medicine, Haukeland University Hospital, Bergen, Norway; 5 Department of Radiology, Haukeland University Hospital, Bergen, Norway; 6 Department of Pathology, Haukeland University Hospital, Bergen, Norway; 7 Department of Respiratory Medicine, Oslo University Hospital, Oslo, Norway; 8 Institute for Clinical Medicine, University of Oslo, Oslo, Norway; 9 Department of Dermatology, Haukeland University Hospital, Bergen, Norway; 10 Centre for International Health, Department of Global Public Health and Primary Care, University of Bergen, Bergen, Norway; 11 Department of Thoracic Medicine, Haukeland University Hospital, Bergen, Norway

**Keywords:** cutaneous vasculitis, lung transplant, reactivation, varicella pneumonia, varicella-zoster virus

## Abstract

**Background.** Varicella-zoster virus (VZV) is a human neurotropic virus which commonly causes infection during childhood, presenting as chickenpox. Later in life it may reactivate as herpes zoster. We report a rare manifestation of reactivation of VZV infection presenting as cutaneous vasculitis and varicella pneumonia in a lung transplant recipient.

**Case presentation.** A 65-year-old man was lung transplanted bilaterally for emphysema and had repeated posttransplant chest infections and colonization with *Pseudomonas aeruginosa*. Nine months post-transplant he presented with dyspnoea and a cutaneous vasculitis-like eruption with a predilection over face, thorax and distal extremities. Initially, VZV reactivation was not suspected due to absence of the typical vesicular eruptions. The diagnosis was confirmed by VZV PCR from the swabs of the ulcer after skin punch biopsy of a lesion and from bronchoalveolar lavage (BAL). The histology of skin biopsy demonstrated epithelial damage and vascular damage but no typical epithelial virus associated changes. The patient responded to antiviral therapy with total remission of rash and VZV DNA was finally not detectable from repeated BAL after 29 days of therapy. However, the pulmonary radiological features and dyspnoea persisted due to reasons possibly unrelated to the VZV infection.

**Conclusion.** Had it not been for the patient to mention the resemblance of the vasculitic rash with his primary VZV infection, the diagnosis would easily have been overlooked. In this case, the biopsy did not show typical histopathologic findings of VZV-vasculitis. What led the diagnosis was a PCR from the wound swab taken after the punch biopsy. This case serves as a reminder for atypical presentation of common conditions in immunosuppressed patients and that extensive diagnostic sampling may be warranted in this group.

## Data Summary

Data was directly drawn from our hospitals’ journal system (including radiology and lab).

## Background

Varicella is an acute infectious disease caused by the varicella-zoster virus (VZV), a DNA virus, member of the herpesvirus group. In the immunocompetent hosts, primary infection often occurs in childhood, as an acute febrile vesicular rash with a favourable prognosis (chickenpox). VZV remains dormant in sensory nerve ganglia, and reactivation of latent infection may occur later in life usually as herpes zoster in the innervated area [1]. In immunocompromised hosts, primary infection or reactivation is often associated with other organ affection such as pneumonia and central nervous system (CNS) infections with a poorer prognosis [2]. In organ transplant recipients the typical vesicular eruptions at onset may be absent. Thus, fatal outcomes have been reported due to delayed diagnoses in this group of patients [2,5]. We report a case of a lung transplant recipient who developed cutaneous vasculitis and pneumonia due to reactivation of VZV. There was a delay in diagnosis as the generalized skin eruption was atypical, lacking the classical vesicles. The diagnosis of VZV reactivation was supported by the positive PCR swab from a punch biopsy taken from a wound lesion and later positive PCR of bronchoalveolar lavage (BAL).

## Case presentation

A 65-year-old man was admitted to our hospital due to a generalized exanthema. Nine months prior he had undergone a bilateral lung transplantation due to severe eosinophilic asthma with progression to chronic obstructive pulmonary disease (COPD), emphysema and respiratory failure. Prior to transplantation he suffered repeated episodes of pneumonias. His lungs were colonized with multidrug-resistant *Mycobacterium intracellulare* 10 years prior to transplantation. Two eradication attempts had been unsuccessful. However, he had spontaneous remission 3 years after the last eradication attempt, and sputum remained negative for *M. intracellulare* thereafter. He also had repeated pneumonias due to *Pseudomonas aeruginosa* and *Achromobacter xylosoxidans,* and multiple eradication attempts were unsuccessful. Therefore, he was given treatment with meropenem before and after the transplantation to suppress the infection.

The transplantation surgery went well without major complications. Forced expiratory volume in 1 s (FEV1) was satisfactory at 2.8 l post-transplantation and he was discharged with a standard prophylactic regime consisting of valganciclovir and trimethoprim-sulfamethoxazole in addition to prednisolone, ciclosporin and mycophenolate as immunosuppressants. He was readmitted with acute dyspnoea 8 weeks after transplantation. Computer tomography (CT) of the chest showed centrilobular nodules with a ‘tree-in-bud’ pattern and bronchial thickening and bronchoscopy revealed copious amounts of thick secretions. Transbronchial biopsy showed no signs of acute rejection. No infectious agent was found in the BAL, only *Candida albicans* in small amounts. As all other findings were compatible with an infectious cause, he was treated with meropenem followed by oral trimethoprim-sulfamethoxazole.

During the following months, the patient was readmitted several times due to dyspnoea and reduced FEV1. Although chronic lung rejection (termed chronic lung allograft dysfunction; CLAD) was not proven, azithromycin was started as a low-dose regimen for immunomodulation as well as short courses of high-dose steroids. Valganciclovir given routinely for CMV-prophylaxis was discontinued approximately 5 months after transplantation due to leucopenia – and 4 months before the patient presented at our unit with the current clinical picture. On several occasions, *Pseudomonas aeruginosa* was cultivated from the BAL and he responded favourably to treatment with anti-pseudomonal therapy. Approximately 7 months after transplantation, a pseudomonal suppression regimen was started with colistin inhalations in addition to ciprofloxacin orally.

**Fig. 1.**
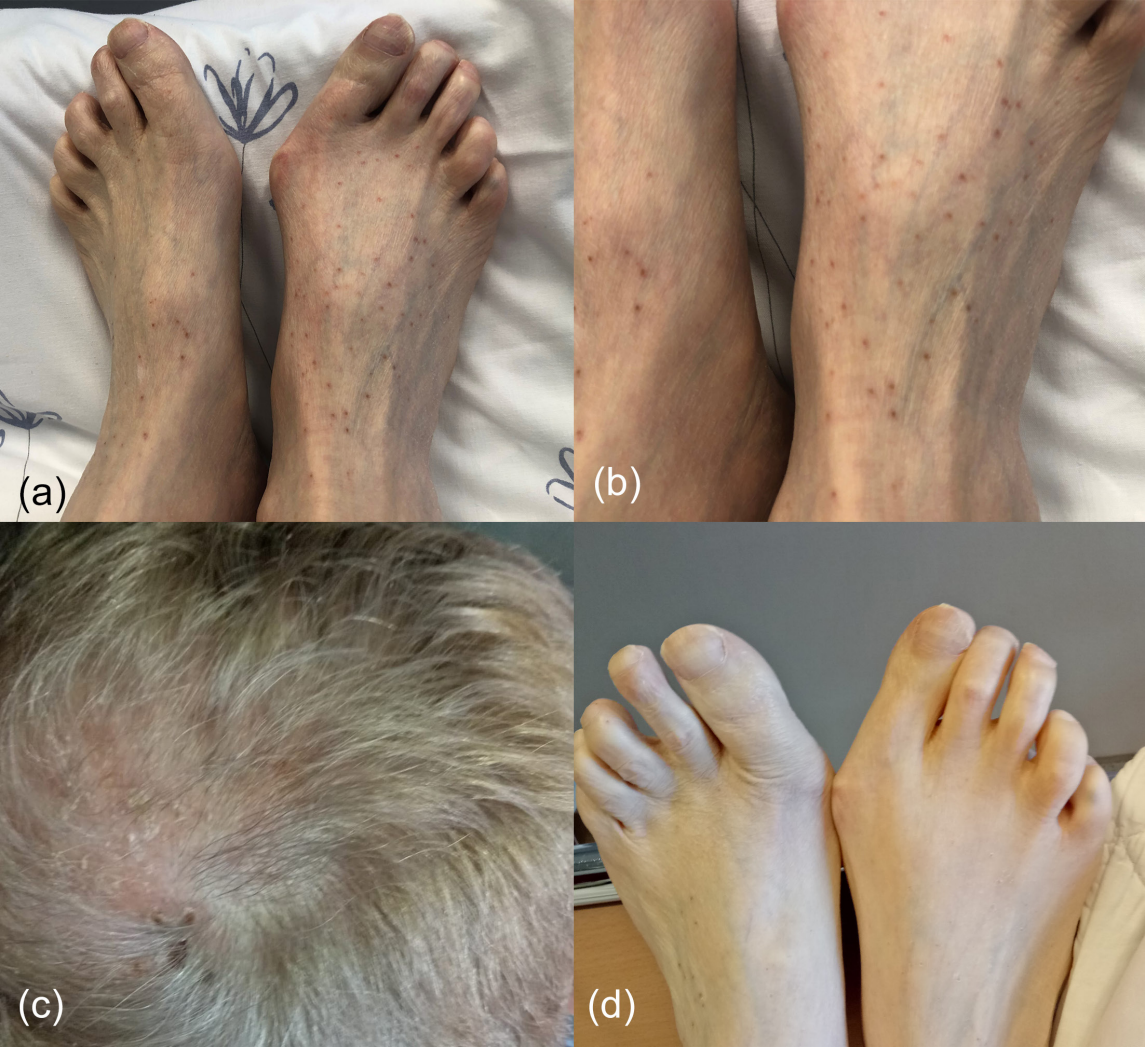
(a), (b) The patient presented with a generalized purpuric and vasculitic-like rash on day 1. (c) One week prior to admission, the patient had presented with painful scalp lesions, interpreted and treated as solar keratoses. Retrospectively we suspect this to have been herpes zoster as the first manifestation of his VZV reactivation. (d) Remission of rash after 2 days of treatment with prednisolone and antihistamines.

**Fig. 2.**
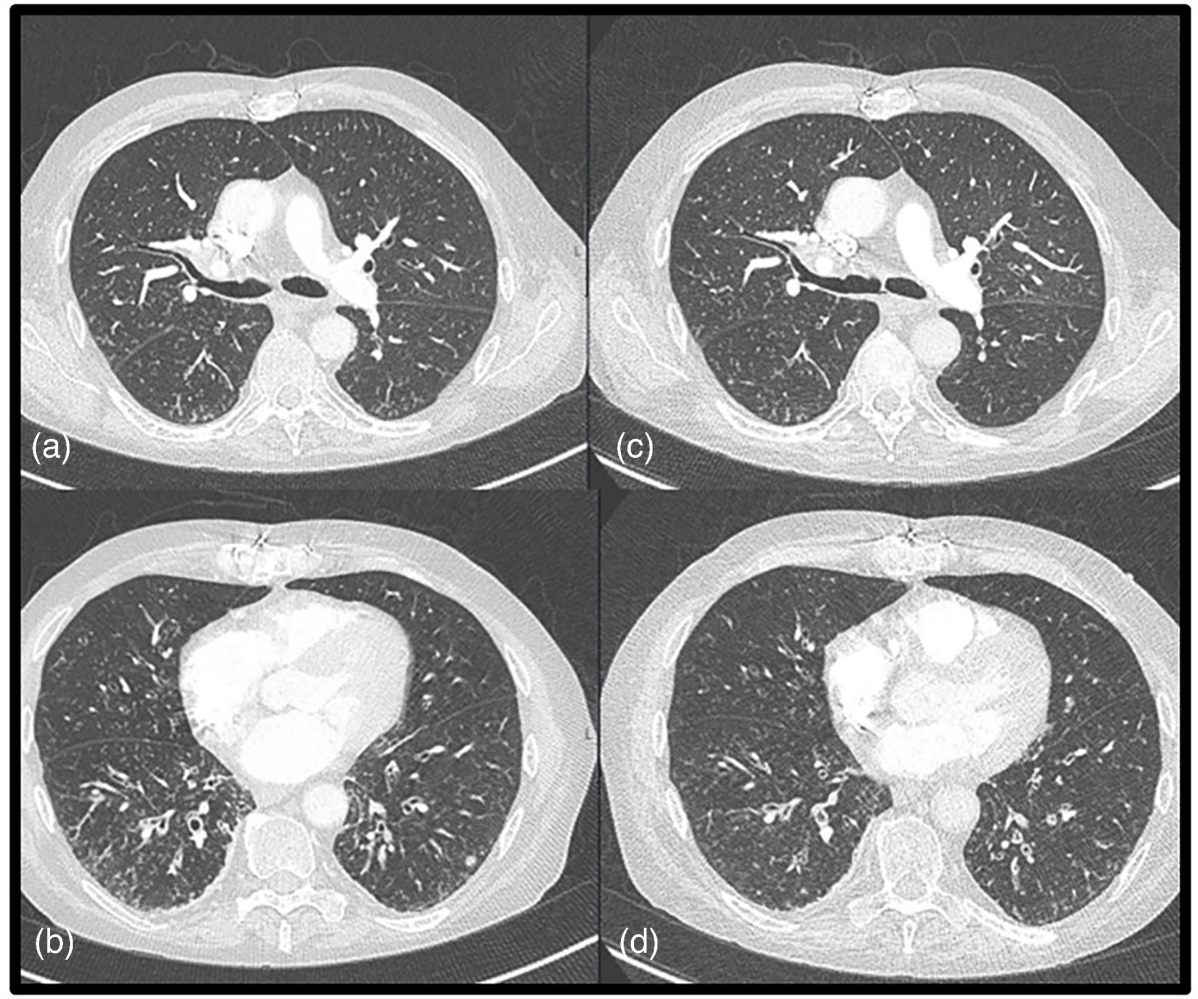
Axial CT images in lung window. (a),(b) (left panels) On admission, small airway changes suggest bronchiolitis; bronchial wall thickening and ‘tree in bud’ changes most pronounced in lower lobes. (c),(d) (right panel) After therapy, slightly less pronounced bronchiolitis changes, but no significant difference.

Two days after initiation of ciprofloxacin, the patient presented to our emergency department with a generalized pruritic exanthema (Fig. 1a and b) and dyspnoea. A week prior, he had received treatment for solar keratosis of the scalp with cryotherapy and betamethasone cream (Fig. 1c). Despite the dyspnoea, his objective respiratory parameters were within normal range and chest radiographs did not show new lesions. CT pulmonary angiogram (CTPA) was nevertheless performed to exclude pulmonary embolism. It showed bronchial wall thickening, multiple small patchy centrilobular nodular opacities with a ‘tree-in-bud’ pattern indicating bronchiolitis and peripheral/subpleural atelectasis secondary to plugged bronchial branches (Fig. 2). The distribution of skin lesions was remarkable, with a predilection of face and thorax in addition to distal extremities, and only sparse lesions on the abdomen. The patient himself pointed out the resemblance to when he had VZV primary infection as a teenager. However, there were no signs of vesicles typical for VZV rash, hence, VZV infection was not considered as an initial diagnosis. An allergic reaction to trimethoprim-sulfamethoxazole was suspected, although the exanthema did not show typical resemblance to a sulfa-reaction either. Trimethoprim-sulfamethoxazole was discontinued, and oral cetirizine was started. Consultation from a dermatologist was sought, and a punch biopsy of the rash was taken. At this point, ciprofloxacin-induced vasculitis was suspected [6] due to temporal relationship with ciprofloxacin initiation and the appearance of rash. Consequently, ciprofloxacin was discontinued and prednisolone increased from the baseline maintenance dose of 10 to 30 mg once daily. This led to quick improvement of pruritus and almost complete remission of the rash within 2 days (Fig. 1d). Antiviral medication was withheld as ciprofloxacin-induced vasculitis was still considered plausible, but VZV-serology was ordered. The VZV serum IgM and IgG were markedly elevated (IgG 3203 IU/ml and IgM >2,3 IU/ml – threshold for positive test >180 IU/ml and >1,3 IU/ml respectively). VZV IgG had been positive pre-transplantation, so there was no suspicion of primary varicella infection. As there were no vesicles to sample, a swab-specimen for detection of VZV DNA was taken from the wound after punch biopsy. Importantly, the swab sample was strongly positive for VZV DNA with a cycle-threshold (CT) value of 24. Consequently, the diagnosis was revised to VZV-reactivation, and treatment was initiated with intravenous aciclovir 10 mg/kg × three daily.

**Fig. 3.**
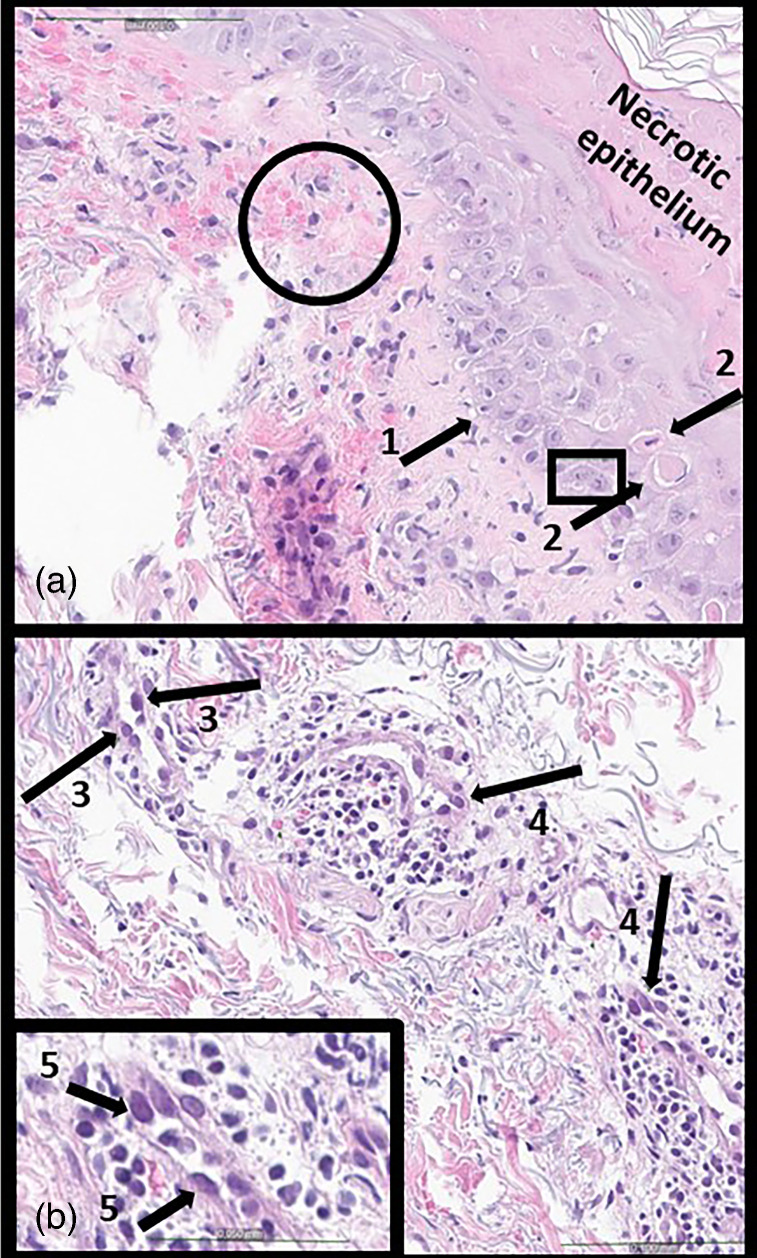
Histopathological features of the punch biopsy of skin affected by pruritic exanthema in a lung transplant patient. (a) The epidermis and the superficial dermis are shown. The arrow (1) shows the interfase damage at the basal layer of the epithelium. The fibrotic dermis is covered by the span of the black arrow. The arrows (2) show two dyskeratinocytes in the epidermis, while the nearby square shows an atypical looking keratinocyte, with a big nucleus and prominent nucleoli. On top the epidermis rest of necrotic epithelium can be seen in a thick eosinofilic layer (right upper corner). Circled are extravasated erythrocytes in the hyaline superficial dermis.) shows dilated capillaries with big nuclei that protrude in the vascular lumina (arrows 3). Around the vessels lymphocytes and few eosinophil granulocytes can be seen and arrows (4) point to erythrocytes outside lumina of damaged capillaries. The arrows (3) indicate in particular nuclei with glossy chromatin and in two of those also eosinofil inclusions can be seen. The appearance of one such nuclei (arrows 5) can be better appreciated in the magnified inlet lower in the picture that represent the vessel far to the right in the picture.

Histopathological examination of the skin punch biopsy showed signs of superficial dermal vascular damage, with perivascular erythrocytes and a moderate perivascular infiltrate of lymphocytes and macrophages, eosinophils, and few plasma cells (Fig. 3). The superficial dermis showed fibrosis and a hyaline appearance; lichenoid damage, some atypical keratinocytes with enlarged nuclei containing prominent nucleoli, and a thick layer of necrosis on the surface surmounted by basket-weave hyperkeratosis were observed in the epidermis (Fig. 3a). A descriptive diagnosis of lichenoid damage, epidermal necrosis and vasculitis was released. On reexamination of the biopsy the absence of typical cytopathic changes in the epithelium of the epidermis or cutaneous adnexal structures was confirmed, but enlarged endothelial nuclei with glassy chromatin, hyperchromasia and eosinophilic vacuole in few nuclei was observed (Fig. 3b, inlet). Unfortunately, the punch biopsy could not be tested for VZV DNA by PCR due to formalin fixation and paraffin embedding, and immunohistochemical analysis for VZV antigens was not available.

Plasma and BAL were also analysed for cytomegalovirus (CMV) DNA, plasma for Epstein–Barr virus (EBV) DNA, hepatitis B virus (HBV) DNA and BK polyomavirus (BKPyV) DNA – all were negative. BAL was cultured and no bacterial growth was detected other than what was interpreted as normal respiratory flora, and an insignificant amount of *Candida albicans*. Notably, there was no growth of pseudomonas species or mycobacteriae.

However, after the initiation of treatment, the patient experienced increasing dyspnoea. A second CT scan of the chest showed the same tree-in-bud-opacities and bronchial wall thickening as previously described a month prior with no signs of worsening. There were no radiological signs typical for virus infection such as bilateral ground glass opacities, septal thickening creating a ‘crazy paving’ pattern or loose consolidations. Copious secretions were observed on bronchoscopy. At this point, the BAL was positive for VZV DNA by PCR. There was no growth of bacteria or fungi, and VZV pneumonia was suspected.

At our hospital, aciclovir is co-administered with intravenous fluids (usually 250–500 ml per infusion). This led to fluid overload, pulmonary oedema and worsening of dyspnoea. The treatment was therefore changed to valaciclovir tablets. After 29 days of antiviral treatment, follow-up BAL did no longer detect VZV-DNA by PCR. However, follow-up CT scan did not show any improvement of the radiological findings (Fig. 2). The treatment was stepped down to a prophylactic dose of valaciclovir for 3 months following discharge. No leucopenia was observed.

The exanthema of the scalp initially interpreted as solar keratosis was retrospectively attributed to herpes zoster. Both the herpes zoster and the generalized vasculitis-like VZV-eruption subsided and there has been no relapse 24 months after treatment.

## Discussion and conclusions

This case illustrates how the transplanted, immunosuppressed patient can present with atypical features of a common disease. The herpes-zoster-manifestation was first misdiagnosed as solar keratosis, and the nodular lung opacities with a tree-in-bud pattern were considered attributable to the previous episodes of *Pseudomonas aeruginosa* pneumonia. There is a possibility that some of the radiological findings could represent bronchiolitis obliterans syndrome, or other specific transplant related disease [7], such as azithromycin responsive allograft dysfunction or neutrophilic reversible allograft dysfunction, since there was no radiological improvement until 2 months after discharge despite successful treatment of the VZV-reactivation. Notably, the transbronchial biopsy findings did not show any signs of allograft rejection and were consistent with unspecific inflammation. The studies on the radiological patterns of VZV pneumonia are scarce [8,11], but describes two predominant imaging patterns: crazy paving (combination of ground glass and septal thickening) and nodular pattern [10]. However, similar patterns are also seen in pneumonias caused by other viruses of the herpes family challenging the radiological confirmation. In our patient the radiological pattern could not discern aetiology.

CNS VZV-vasculitis and vasculopathy are known entities [12]. However, there are relatively few reports of cutaneous vasculitis alone or in combination with other organ involvement in immunocompetent and immunocompromised patients [12,16]. To our knowledge, this is the first report of a combined cutaneous vasculitis and pneumonia due to VZV in a lung transplant recipient. In our case, the diagnosis of VZV-reactivation was regarded as highly unlikely as the generalized skin eruption was atypical, completely lacking the classical vesicles typical of VZV skin manifestations. Also, a very quick improvement of the vasculitic eruption within 48 h after initiation of steroids, favoured the initial diagnosis of ciprofloxacin-induced vasculitis [6]. The punch biopsy did not show the typical epithelial changes associated with VZV either, although the epidermal necrosis could have raised a suspicion of VZV infection, and vasculitis with viral cytopathy is not reported as a common finding. Indeed, vasculitis may be the only manifestation of VZV in an otherwise unspecific finding of florid lymphocytic inflammation that can at times appear pseudolymphomatous [17].

After finding markedly elevated IgG and IgM in serum, a positive VZV-PCR from a skin lesion, and a strongly positive VZV-PCR from BAL – there was so much objective evidence of a generalized VZV-reactivation that the original diagnosis of ciprofloxacin-induced vasculitis had to be rejected.

A raised VZV-DNA in plasma was, however, never detected, which is perhaps remarkable regarding the widespread nature of the reactivation. However, our hospital’s method for detection of VZV is not validated for plasma.

CMV reactivation is a well-known risk in the solid organ and haematopoietic stem-cell-transplanted patient and thereby routinely screened for and/or treated with antiviral prophylaxis in the post-transplant period. The implications of clinical and subclinical VZV reactivation are not investigated to the same degree, and one may wonder if some of these cases go undetected as screening methods for detection of VZV in the blood are limited as compared to CMV.

To what extent the diagnosis and treatment of this VZV-reactivation has influenced our patients long-term post-transplant prognosis is unknown. Nevertheless, this case report may serve as a reminder to keep an open diagnostic mind especially regarding the deeply immunosuppressed patient, making sure diagnosis is correct through invasive sampling – and not rejecting diagnoses even in the setting of atypical presentations.

The patient has been informed and consents to the publication of this case report.

## Abbreviations

CLAD: chronic lung allograft dysfunction
CNS: central nervous system
COPD: chronic obstructive pulmonary disease
CT: computer tomography
CTPA: CT pulmonary angiogram
FEV1: forced expiratory volume in one second
PCR: polymerase chain reaction
VZV: varicella-zoster virus
